# The efficacy and safety of continuous versus single-injection popliteal sciatic nerve block in outpatient foot and ankle surgery: a systematic review and meta-analysis

**DOI:** 10.1186/s12891-019-2822-7

**Published:** 2019-10-10

**Authors:** Hsuan-Hsiao Ma, Te-Feng Arthur Chou, Shang-Wen Tsai, Cheng-Fong Chen, Po-Kuei Wu, Wei-Ming Chen

**Affiliations:** 1 Department of Orthopaedics and Traumatology, Taipei Veterans General Hospital, No. 201, Sec 2, Shi-Pai Road, Taipei, 112 Taiwan; 2 Department of Orthopaedics, School of Medicine, National Yang-Ming University, Taipei, Taiwan

**Keywords:** Complication, Continuous popliteal sciatic nerve block, Foot and ankle surgery, Outpatient surgery, Pain management

## Abstract

**Background:**

Continuous popliteal sciatic nerve block (CPSNB) has been performed in outpatient foot and ankle surgery as a regional anesthesia method to relieve postoperative pain. Its efficacy as well as safety is yet to be established. There are two purposes of this study: (1) to validate the efficacy of CPSNB with regards to better pain relief and reduced analgesics consumption; (2) to assess the safety of CPSNB.

**Methods:**

We performed a comprehensive literature review on Web of Science, the Cochrane Library, PubMed and Embase and only included randomized controlled trials (RCTs). Five RCTs that compared the efficacy and safety of CPSNB with the single-injection popliteal sciatic nerve block group were included. The primary outcome parameters were visual analog scale (VAS) scores at postoperative 24, 48 and 72 h. The secondary outcome parameters were amount of oral analgesics consumed, overall patient satisfaction and need of admission after surgery. A sensitivity analysis was performed to explore the consistency of the results.

**Results:**

In comparison with the single-injection group, CPSNB was associated with a lower VAS score at postoperative 24 and 48 h (*p* < 0.05). There were no neuropathic symptoms or infection events after the nerve block. However, there were several minor complications associated with the pump and catheter system, with drug leakage being the most common complication (*N* = 26 of 187, 13.9%).

**Conclusion:**

CPSNB is an effective method in pain management for outpatient foot and ankle surgery. Both methods appear to be safe as none of the patients experienced neuropathic symptoms or infection. Further studies with larger sample size are needed to compare the risk of major complications between the two methods.

**Level of evidence:**

I; meta-analysis.

## Introduction

Foot and ankle surgery is increasingly performed in a same-day, outpatient setting but is associated with moderate to severe postoperative pain which may lead to prolonged post-anesthesia care unit (PACU) stay, unanticipated admission and decreased patient satisfaction [1–3]. Postoperative pain was one of the most common reasons for unanticipated admission, in which 57.6% of them were orthopaedic patients [4]. This moderate to severe postoperative pain following foot and ankle surgery can persist up to postoperative day 3 [5], which single-injection popliteal sciatic nerve block may not be adequate to manage the pain [6]. Therefore, continuous popliteal sciatic nerve block (CPSNB) has been developed as a potential pain management method. Several randomized controlled trials have been conducted to compare the efficacy and complications of CPSNB versus single-injection nerve block [7–11]. Some studies concluded that CPSNB was more effective in pain management than the single-injection nerve block [7, 9–11], while Elliot et al. suggested that it was still debatable whether the additional benefits of CPSNB are worthy of its extra time and cost involved because the pain scores were very low in both groups [8]. Therefore, the first aim of this meta-analysis was to determine the efficacy of CPSNB compared with single-injection group, as a postoperative pain management in patients who had undergone foot and ankle surgery.

In addition to the efficacy, safety concerns are another important issue that should be validated. Neuropathic symptoms and infection following the nerve block are two major complications mentioned in current literature [12–16]. The incidence of neuropathic symptoms after CPSNB has been reported in some studies to be relatively low [13, 15], but recent studies found a higher rate [16–18]. Other complications including accidental falls, adverse drug reaction and other minor complications associated with the infusion system were also mentioned [7–11, 19]. Thus the second aim of this meta-analysis was to assess the safety of CPSNB and to identify major and minor complications associated with CPSNB.

## Materials and methods

### Search strategy

**Fig. 1:**
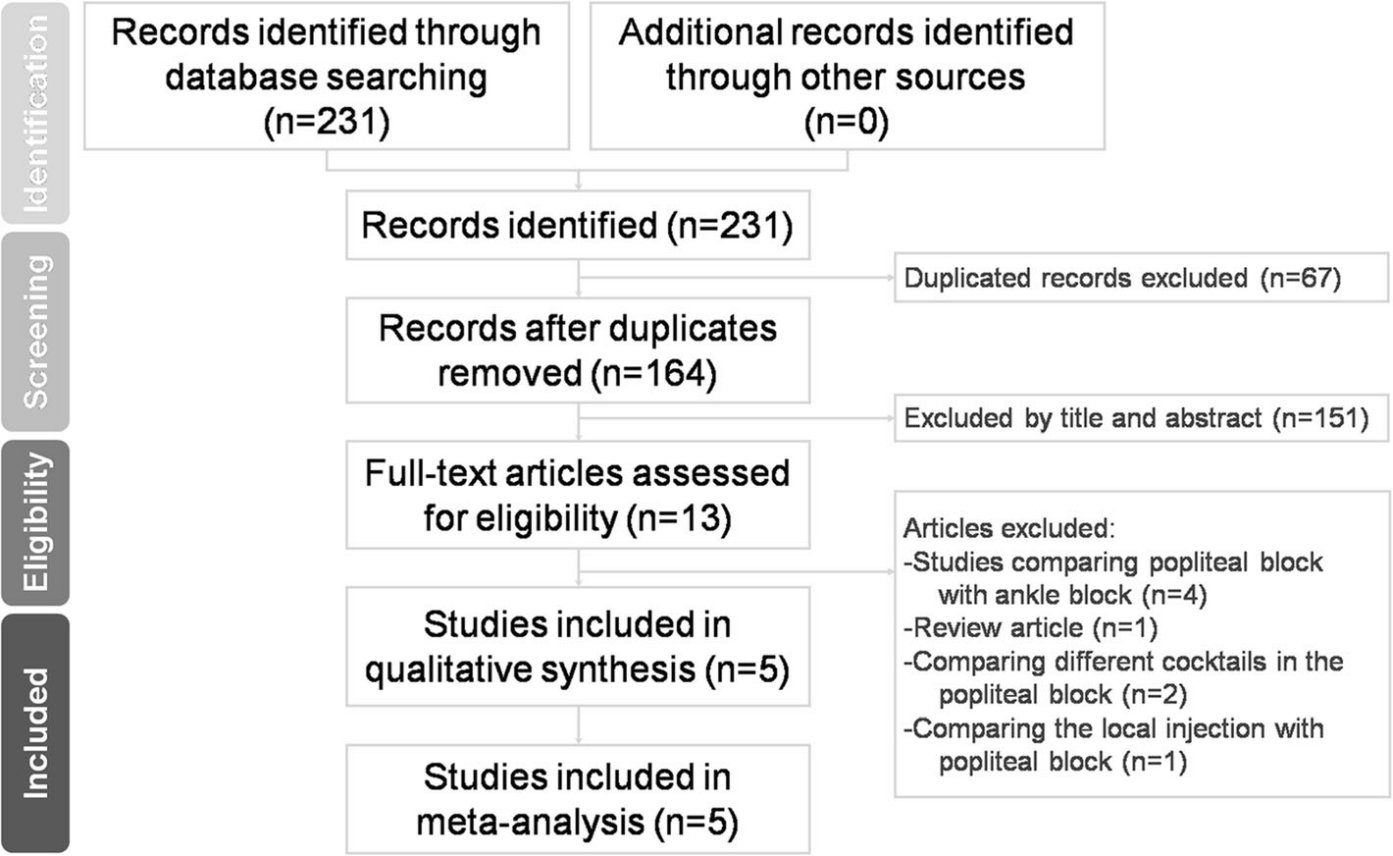
Preferred reporting items for systematic reviews and meta-analysis (PRISMA) flow diagram for the searching and identification of included studies

We searched databases including Web of Science, the Cochrane Library, PubMed and Embase from the earliest record to December 2018, for randomized controlled trials (RCTs) that validated the efficacy and safety for CPSNB in outpatient foot and ankle surgery. We manually reviewed all the bibliographies of the included studies for relevent references. We excluded studies that were not written in English or not available in full text. Our search strategy included the following keywords in various combination: (popliteal block OR regional anesthesia OR nerve block OR popliteal sciatic nerve block) AND (ankle fracture OR calcaneal fracture OR foot and ankle surgery). We enrolled only RCTs and excluded comparative cohort studies, case series and case reports. The included studies should comprise at least two treatment arms: continuous popliteal sciatic nerve block and single-injection popliteal sciatic nerve block. The preferred reporting items for systematic reviews and meta-analysis (PRISMA) guidelines were applied in our search strategy (Fig. 1).

### Inclusion criteria and study selection

All studies that were eligible for our analysis met the PICOS criteria (population, intervention, comparator, outcomes, study design). Population: patients who had undergone outpatient foot and ankle surgery. Intervention: Using continuous popliteal sciatic nerve block as the pain control method. Comparator: Single-injection popliteal sciatic nerve block. Outcomes: Visual analog scale (VAS) at different time points, amount of oral analgesics consumed, patient satisfaction and need of admission. Studies must have a follow-up rate of at least 80%, and at least 1 of the above outcome parameters was included. Study design: interventional randomized controlled trials.

### Data extraction and quality assessment

Two reviewers extracted data from all identified articles using a predetermined form. Information about the first author, year, study design, enrolled sample number, type of treatment arms, anesthesia method, approach of popliteal sciatic nerve block, regimen of infiltration analgesics, type of foot and ankle surgery, outcome parameters to evaluate pain, amount of oral analgesics consumed, patient satisfaction and need of admission after surgery were listed in Table 1. Jadad score was used to assess the quality of the included RCTs. The score ranged from 0 to 5; we defined a score of 4–5 points as good, 2–3 points as fair and 0–1 point as a study with poor methodology. Discrepancies between the two reviewers were solved by discussion.

**Table 1:** Summary of the included studies

Author	study design	G1 (CPSNB)/G2 (SPSNB)	Anesthesia Method	Age G1/G2 (years)	Gender G1/G2 (female%)	Infusion technique	approach	Regieme of first shot	Surgery enrolled	Outcome measurement						Quality assessment^a^
								Regieme of continuous nerve block		a	b	c	d	e	f	
Ding 2015	RCT	23/21	General Anesthesia	51/30	47.8%/52.4%	I-flow On Q pump	posterior/ultrasound-guided	20 ml 0.5% lidocaine with epinephrine 20 ml 0.5% bupivacaine	ankle fractures	v	v	v	v	v		4
								8 ml/hr. 0.2% ropivacaine								
Elliot 2010	RCT	27/27	General Anesthesia	N/A	N/A	Stryker PainPump II	lateral/nerve stimulator	20 ml 0.5% bupivacaine	ankle fusions hindfoot fusions total ankle replacement tendon reconstruction subtalar fusion	v	v	v	v		v	5
								4 ml/hr. 0.25% bupivacaine								
Zaric 2004	RCT	30/30	Spinal Anesthesia	47/46	83.3%/90%	Baxter LV5 elastomeric pump	posterior/nerve stimulator	30 ml 0.5% ropivacaine	hallux valgus correction metatarsal osteotomy great toe arthrodesis	v	v	v	v	v	v	5
								4 ml/hr. 0.25% ropivacaine								
White 2003	RCT	10/10	General Anesthesia	58/50	60%/70%	C-bloc™ PNB system elastometric pump	lateral/nerve stimulator	30 ml 0.25% ropivacaine	ankle or foot procedure	v	v	v	v	v	v	5
								5 ml/hr. 0.25% ropivacaine								
Ilfeld 2002	RCT	15/15	Regional Block	56.1/51.5	66.6%/73.3%	Microject PCA pump	posterior/nerve stimulator	50 ml 1.5% mepivacaine with epinephrine	hallux valgus correction ankle fracture Achilles tendon repair lesser toe correction subtalar fusion tibial reconstruction	v	v	v		v		5
								8 ml/hr. 0.2% ropivacine								

^a^Jadad score; *CPSNB* Continuous popliteal sciatic nerve block, *SPSNB* Single-injection popliteal sciatic nerve block, *N/A* Not applicable

Outcome measurement: a = VAS at 24 h, b = VAS at 48 h, c = VAS at 72 h, d = total amount of oral analgesics at 72 h, e = patient satisfaction, f = postoperative admission

### Statistical analysis

The standardized mean differences (SMDs) of postoperative VAS score at different time points between the CPSNB and single-injection popliteal sciatic nerve block was the primary outcome. The VAS scores from each study at 24, 48 and 72 h were included. A negative SMD value indicated CPSNB to be a favorable treatment option. The secondary outcomes were amount of oral analgesics consumed (assessed with SMD), overall patient satisfaction and need of admission after CPSNB or single-injection popliteal sciatic nerve that were assessed with odds ratio (OR). We used random effect model to pool individual SMDs and ORs. Analyses were performed using Comprehensive Meta-Analysis (CMA) software, version 3 (Biostat, Englewood, NJ, USA). Between-trial heterogeneity was determined by using *I*^*2*^ tests; values > 50% were regarded as considerable heterogeneity. Statistical significance was defined as *p*-values < 0.05. Due to concerns of inconsistency results in this study, the validity and robustness of effect estimates were adjusted accordingly. A sensitivity analyses was performed by excluding the one study which contained the smallest sample size of the pooled data. We used Trial Sequential Analysis software (Copenhagen Trial Unit, Copenhagen, Denmark) for the trial sequence analysis. Trial sequence analysis calculates required information size which compares well to a sample size calculation for a RCT according to an overall type I error of 5% and a power of 80% [20]. Two reviewers used Cochrane collaboration’s tool for assessing risk of bias in each included randomized trials. Funnel plots and Egger’s test were used to examine potential publication bias.

## Results

A total of 231 relevant articles were identified according to our search strategy. Sixty-seven duplicate records were removed. One hundred and fifty-one studies were excluded by title and abstract. Based on the inclusion criteria, 8 studies were excluded after reading the full text. Finally, 5 articles that compared the efficacy of CPSNB in outpatient foot and ankle surgery with single-injection popliteal sciatic nerve block were included for our meta-analysis. Baseline characteristics of the 5 included studies are summarized in Table 1. Quality of these five included RCTs were good (Jadad score 4–5 points).

### Meta-analysis results

#### VAS score at 24, 48 and 72 h

**Fig. 2:**
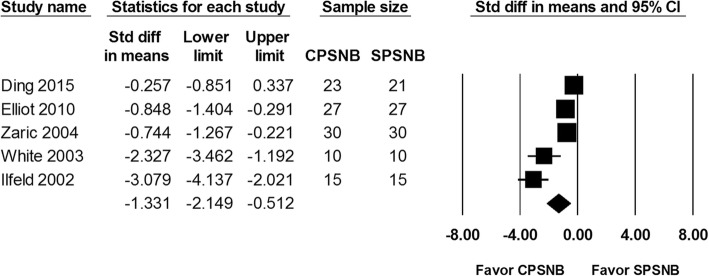
The effect of continuous popliteal sciatic nerve block (CPSNB) on VAS score at 24 h as compared with the single-injection group

**Fig. 3:**
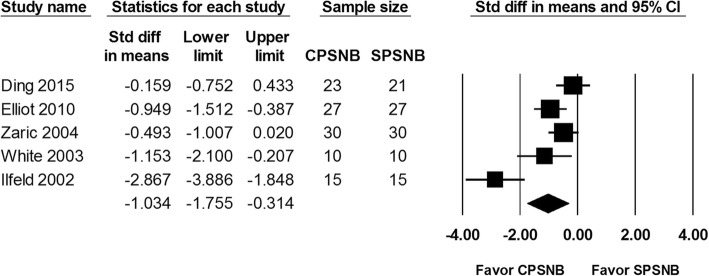
The effect of continuous popliteal sciatic nerve block (CPSNB) on VAS score at 48 h as compared with the single-injection group

**Fig. 4:**
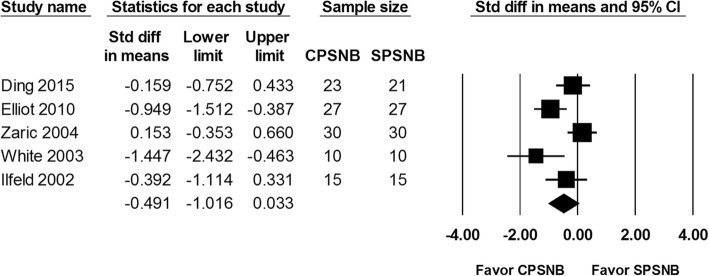
The effect of continuous popliteal sciatic nerve block (CPSNB) on VAS score at 72 h as compared with the single-injection group

The VAS score at 24, 48 and 72 h were reported in 5 studies including a total of 208 patients. The analysis showed a significantly lower VAS score at 24 h in the CPSNB group in comparison with the single-injection group (SMD: -1.331; 95% CI − 2.149 to − 0.512; Heterogeneity: I^2^ = 85.1; Fig. 2). In addition, there was a lower VAS score at 48 h noted in the CPSNB group (SMD -1.034; 95% CI − 1.755 to − 0.314; Heterogeneity: I^2^ = 82.2; Fig. 3). Based on the trial sequence analysis, both methods had adequate sample size to compare the VAS scores at 24 and 48 h (Additional file 1: Figure S1 and Additional file 2: Figure S2). The VAS score at 72 h was not different between the groups (SMD: -0.491; 95% CI − 1.016 to 0.033; Heterogeneity: I^2^ = 69.7; Fig. 4). However, this result was considered inconclusive because of inadequate number of studies according to the trial sequence analysis. (Additional file 3: Figure S3).

#### Total amount of Oral analgesics consumed at 72 h

**Fig. 5:**
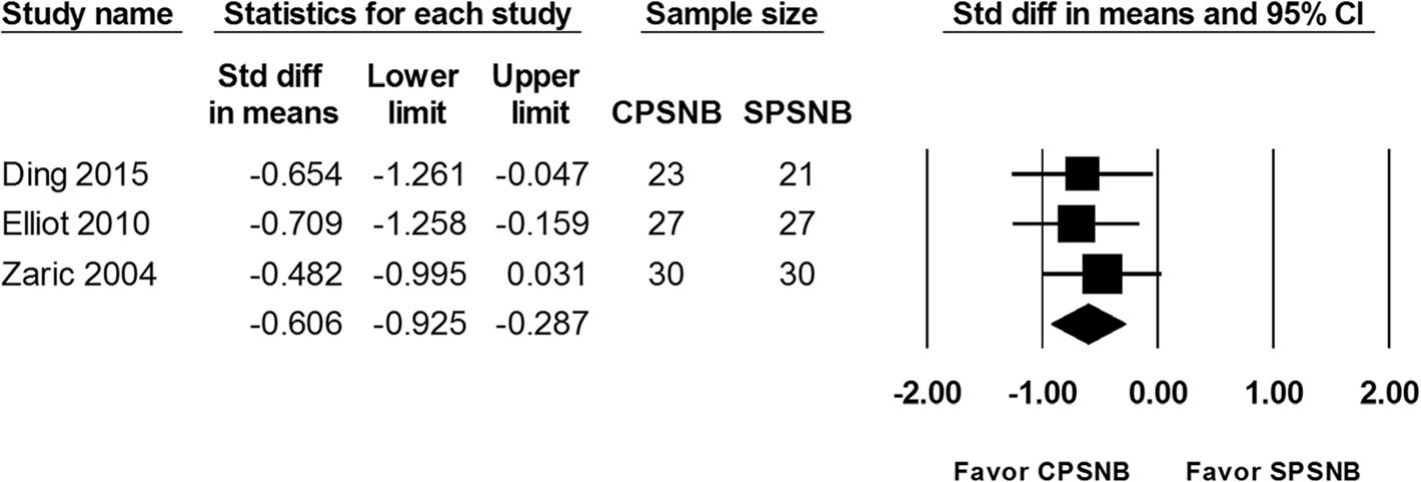
The effect of continuous popliteal sciatic nerve block (CPSNB) on total amount of oral analgesics at 72 h as compared with the single-injection group

Total amount of oral analgesics consumed at 72 h was reported in 4 studies and included 158 patients. The pooled data from patients that received CPSNB was associated with less amount of oral analgesics consumed (SMD: -0.606; 95% CI − 0.925 to − 0.287; Heterogeneity: I^2^ = 0; Fig. 5). However, the enrolled number did not reach required information size. Thus, this result was considered inconclusive. (Additional file 4: Figure S4).

#### Patient satisfaction scale

There were 4 studies that recorded the patient satisfaction scale and included 154 patients. However, we are not able to perform analysis of this outcome domain because patient satisfaction scales used in the 4 studies were inconsistent. Two studies showed a higher degree of satisfaction in the CPSNB group compared with the single-injection group [7, 9], while the other two studies did not find a significant difference [10, 11].

#### Postoperative admission

**Fig. 6:**
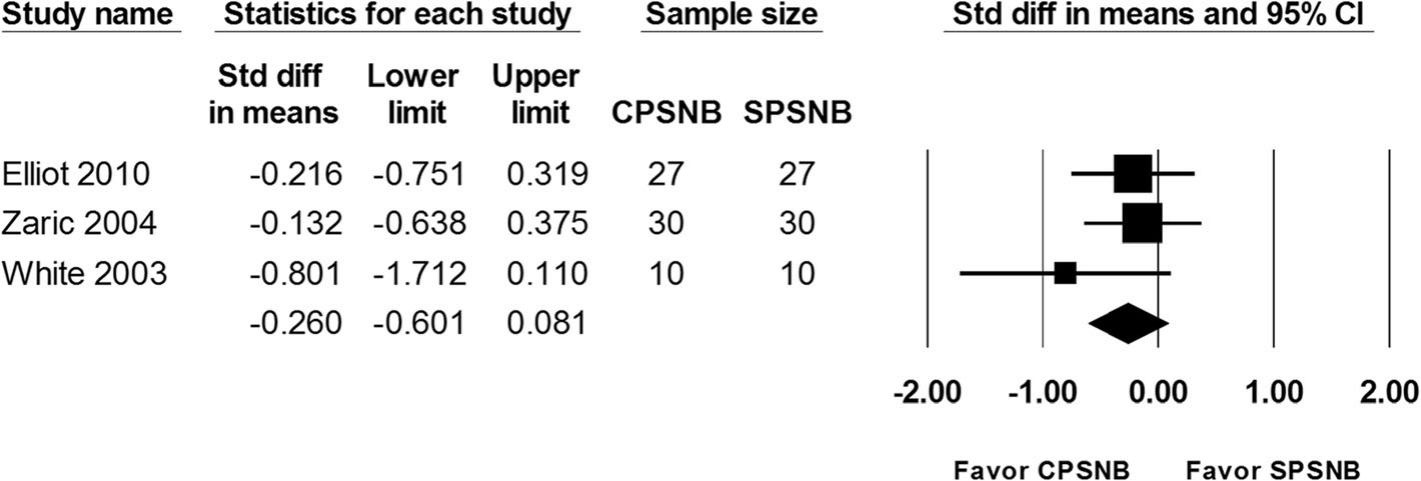
The effect of continuous popliteal sciatic nerve block (CPSNB) on patient postoperative admission with the single-injection group

The postoperative admission rate was reported in 3 studies and a total of 134 patients were evaluated. No significant difference was found between the CPSNB and the single-injection group. (OR: -0.260; 95% CI − 0.601 to 0.081; Heterogeneity: I^2^ = 0; Fig. 6). However, the enrolled number was less than the required information size. Therefore, this result was inconclusive (Additional file 5: Figure S5).

#### Adverse events and complications

The adverse events and complications of the enrolled studies were extracted and listed in Table 2. There were no major complications associated with the nerve block procedure or with infusion system, including neuropathic symptoms, infection, adverse drug reaction or accidental fall. However, there were some minor complications, including drug leakage (*N* = 26 of 187, 13.9%), catheter dislodge (*N* = 9 of 187, 4.8%), pump malfunction (*N* = 4 of 187, 2.1%) and catheter blockade (*N* = 2 of 187, 1.0%).

**Table 2:** Descriptions of complications in the included studies

Author	study design	G1(CPSNB) / G2(SPSNB)	Complications
Ding 2015	RCT	23/21	G1: 5/23 catheter dislodge; 2/23 pump malfunction
Elliot 2010	RCT	27/27	All: 6/54 drug leakage, 2/54 catheter blockade, 1/54 catheter dislodge, 1/54 pump malfunction G1:0/27 adverse drug reaction G2:1/27 numbness on foot dorsum for 5 days, recovered
Zaric 2004	RCT	30/30	All: 5/60 drug leakage,5/60 difficult adaptation to the device, 1/60 catheter dislodge, G1: 0/30 adverse drug reaction
White 2003	RCT	10/10	G1:8/10 “tingling sensation” in the foot G2:1/10 “tingling sensation” in the foot
Ilfeld 2002	RCT	15/15	All: 15/30 drug leakage, 9/30 nonscheduled contact with the physician, 2/30 catheter dislodge, 1/30 pump malfunction

*CPSNB* Continuous popliteal sciatic nerve block

*SPSNB* Single-injection popliteal sciatic nerve block

#### Publication bias

**Fig. 7:**
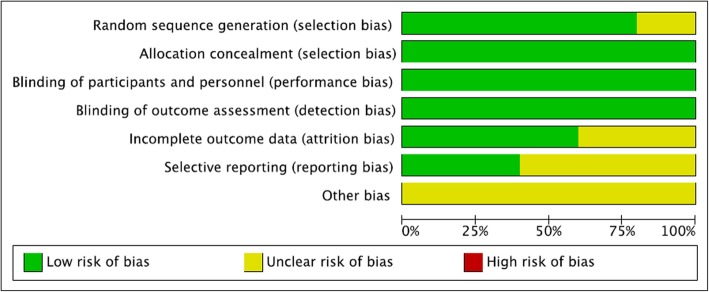
Summary of the assessment of the risk of bias

**Fig. 8:**
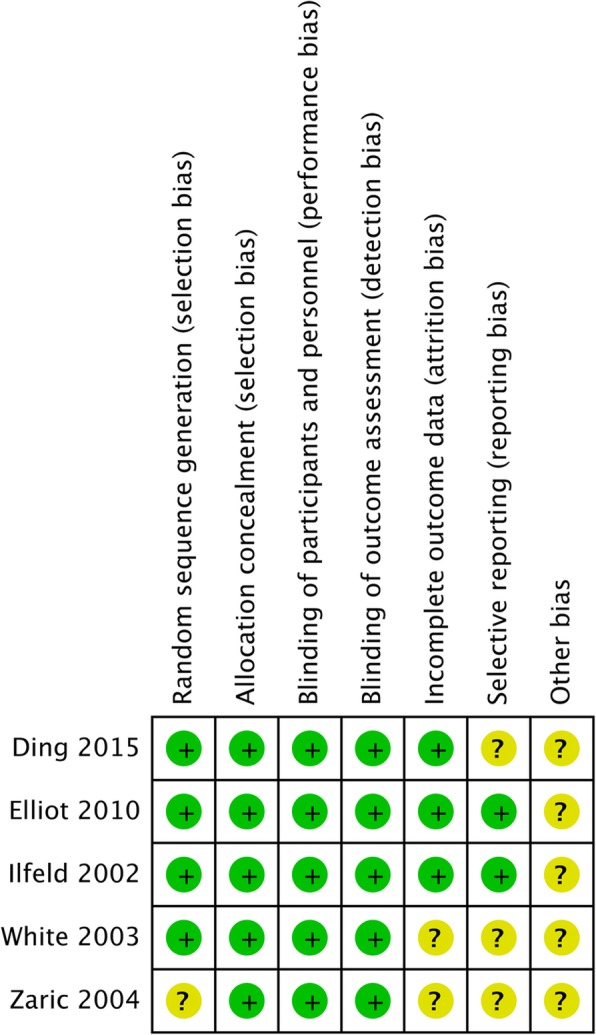
Results of risk of bias evaluation for each study according to the recommendations of the Cochrane Collaboration

The results of the risk of bias evaluation for each study is summarized in Fig. 7 and Fig. 8. The allocation concealment bias (selection bias) was regarded as low risk. The completeness of the reported data (reporting bias) was unclear in 3 of the 5 (60%) studies included in this analysis. The Egger’s test revealed no significant publication bias regarding the overall SMD and odds ratio for included outcome parameters. The funnel plots for SMD and log odds ratio of all of the outcome parameters from each study are shown in Additional file 6: Figure S6, Additional file 7: Figure S7, Additional file 8: Figure S8, Additional file 9: Figure S9, Additional file 10: Figure S10.

#### Sensitivity analyses

A similar result was obtained by using the random-effect model. The SMD and OR were analyzed after excluding one study which contained the smallest sample size. The analysis showed consistent results of outcome parameters in the CPSNB group in comparison with the single-injection group including VAS score at 24 h (SMD: -1.121; 95% CI − 1.969 to − 0.274), VAS score at 48 h (SMD: -1.022; 95% CI − 1.877 to − 0.167), VAS score at 72 h (SMD: -0.327; 95% CI − 0.823 to 0.168), total amount of oral analgesics consumed at 72 h (SMD: -0.554; 95% CI − 1.092 to − 0.276) and postoperative admission (OR: -0.172; 95% CI − 0.540 to 0.196).

## Discussion

In this meta-analysis, we focused on the efficacy of CPSNB compared with that of single-injection popliteal sciatic nerve block in outpatient foot and ankle surgery. We included 5 RCTs with 208 patients. In comparison with the single-injection nerve block group, patients who received CPSNB presented with less pain (VAS score at 24 and 48 h). Outcome domains including amount of oral analgesics consumed at 72 h, VAS score at 72 h, rate of postoperative admission were considered inconclusive according to the results of trial sequence analysis. There were no major complications including neuropathic symptoms and infection reported in these 5 studies. However, there was a substantial rate of catheter or pump associated complications including catheter dislodge, leakage, irritation, block and pump malfunction.

Effective pain management using regional nerve block as an analgesic adjuvant to anesthesia allows surgeons to perform foot and ankle surgeries as outpatient procedures. Its potential benefits include improved pain control, better patient satisfaction, reduced postoperative opioid consumption and lower costs [21–23]. In the setting of an outpatient procedure, efforts have to be made to prevent unanticipated admissions. In a prospective study of patients who had undergone various ambulatory surgeries, pain was accounted for 12% of unplanned admissions in which 57.6% of them were orthopedic patients [4]. The analgesic effects of a single-injection nerve blockade is limited to 15–22 h [6], which might not be adequate to relief severe pain following foot and ankle surgery up to postoperative day 3 [5]. Therefore, patients might benefit from continuous nerve block to have sustained pain relief.

This study was the first meta-analysis to validate efficacy and safety of CPSNB after foot and ankle surgery as an outpatient procedure, which included five randomized controlled trials for the analysis [7–11]. Compared with the single-injection group, CPSNB was associated with better pain relief up to 48 h. The duration of better pain relief was compatible with the duration of drug administration. In these five studies, a catheter was placed for 48 to 72 h after surgery [7–11]. Difference of VAS score between the continuous and single-injection popliteal sciatic nerve block group might also be diminished as the post-surgical pain decreases with time. In addition, Sutton et al. and Landorf et al. have previously determined the minimal clinical important difference (MCID) of foot and ankle surgeries. The authors determined that a difference in VAS score of 0.8 can be considered the MCID [24, 25]. In our study, the difference between two groups in VAS score at 24 h and 48 h were 2.28 points and 1.18, respectively. This further confirms that CPSNB resulted in improved pain management in these patients.

Despite the benefits of better pain control and less analgesics consumption, several inherent risks have to be assessed with popliteal sciatic nerve block and the continuous perineural local anesthetic infusion system. Neuropathic symptoms is one of the major complication associated with popliteal sciatic nerve block that raise the concerns [12–14]. Compére et al. prospectively enrolled 400 patients who received CPSNB. Three major complications were reported, including two neuropathies (0.5%) [13]. Meanwhile, the incidence of neuropathic symptoms after popliteal sciatic nerve block were higher in recent studies [16–18]. Park et al. conducted a retrospective study of 827 patients evaluating the safety of popliteal sciatic nerve block. Twenty-two (2.7%) developed neuropathic symptoms secondary to the nerve block and 7 (0.8%) patients had unresolved symptoms at the final follow-up visit [17]. The concern of neuropathic symptoms after popliteal block was supported by the results from Anderson et al., a retrospective study of 1014 patients who received single-injection or continuously popliteal sciatic nerve block. One hundred and thirty-five (13.3%) patients developed varying neuropathic symptoms, while most of them (*N* = 119, 87.4%) reported exclusively sensory deficits. Of the 99 patients who received a continuous infusion with a catheter, 17 (17.1%) patients developed neuropathic symptoms and 7 (7.0%) of them were likely related to the popliteal block. These neuropathic symptoms required an average of 58.6 days to resolve, while one of them remained unresolved up to 8 months [18]. In a prospective cohort study conducted by Gartke et al., the incidence of neuropathic symptoms after the CPSNB was even higher as 41% of patients at postoperative 2 weeks and 24% at 34 weeks had persisted symptoms [16]. Most of the neuropathic symptoms described were sensory deficits of various degrees. The varied incidence of neuropathic symptoms might be due to different study designs (prospective or retrospective data collection), the definition of neuropathic symptoms, and the frequency and duration of follow-up visits [13, 16–18].

Regarding risk factors for neuropathic symptoms, Anderson et al. did not find an association between neuropathic symptoms with smoking, diabetes, tourniquet location or time, use of steroids and/or epinephrine, single or continuous block, and use of ultrasound or nerve stimulator. There was a younger average age in patients who had neuropathic symptoms (47.3 ± 15 years vs. 50.2 ± 17.2 years, *P* = 0.039), compared with those who did not. A higher average tourniquet pressure was found in patients who had neuropathic symptoms (309.4 ± 28.9 mmHg vs. 303.4 ± 24.9 mmHg, *P* = 0.013). Although a statistical significance was noted, the authors suggested that this difference might have minimal clinical significance. Gartke et al. found that smoking history was a borderline significant factor for developing neuropathic symptoms (adjusted OR: 2.25, 95% CI: 0.96–5.33). The authors did not find tourniquet time, tourniquet application site, experience of the specialist performing the block, use of prophylactic antibiotics, and type of anesthesia to be associated with neuropathic symptoms. Although the exact mechanism causing these neuropathic symptoms remain controversial, some authors have hypothesized that some anesthetics may be directly toxic to nerve fibers [26–28]. Local anesthetics including ropivacaine and bupivacaine block impulse conduction in nerve fibers through inhibition of both sodium ion and potassium ion channels [29]. In addition, the concentration and duration of local anesthetics have been shown to contribute to chemical neurotoxicity in an animal model [27]. Increased amount of anesthetics was associated with an elevated intrafascicular pressure that exceeds the nerve capillary perfusion pressure, which would lead to ischemic injury. On the other hand, drugs administered along with the anesthetics such as epinephrine can have a local vasoconstrictive effect that potentially aggravates the ischemic injury [16, 18, 26, 28]. Despite these hypothesis, there are no clinical studies to validate the relationship between the use of common local anesthetics or epinephrine and postoperative neuropathic symptoms. Therefore, studies with high level of evidence are warranted to validate the safety of corticosteroids, epinephrine and each anesthetic. For patients with a smoking history or pre-existing neuropathies, an alternative pain control method to CPSNB should be considered to avoid postoperative neuropathic symptoms.

In the 5 RCTs included in this meta-analysis, Elliot et al. reported a patient in the placebo group who experienced numbness on the dorsal foot which resolved after 5 days [8]. White et al. found a higher incidence of “tingling sensation” on the foot in the CPSNB group (80% versus 10% in the placebo group) [10]. The duration of this sensation was not described. Anderson et al. defined abnormal postoperative neuropathic symptoms as burning, pain, numbness, or tingling in the operative limb that persisted for at least 7 days following a single injection or after catheter removal for continuous infusions. According to this definition, there were no neuropathic events in the 5 studies included in this meta-analysis.

Although no major complications were mentioned in either group, there was a significantly higher rate of minor complications associated with the infusion system, including drug leakage, catheter dislodge, pump malfunction and catheter blockade. When using a portable infusion system, patients have to take an extra responsibility that comes with the device. Those minor complications might lead to a significant rate of nonscheduled contact with the physician (*N* = 9 of 30, 30%) [11] and might affect the overall satisfaction rate. A prudent assessment of patients’ desire and a comprehensive verbal and written instruction would be warranted.

There are several limitations that should be recognized. First, the small sample size and a high heterogeneity of clinical settings between studies, including age, gender, doses and regimens used in CPSNB, surgical approaches, catheter locations and types of anesthesia may effect the results, which should be interpreted with caution. Second, we searched only for English articles but not articles in other languages or unpublished data. This might be a potential source for publication bias. Third, there was an inherent flawed setting in these studies that compared CPSNB with the placebo group. A potential bias associated with a “wash out” effect existed in the placebo group. Continuous saline infusion in the placebo group might have diluted the initial, single-injection regional block and shortened the duration. This can lead to a bias toward the efficacy of CPSNB [11]. Finally, several important outcome parameters with regard to safety concerns of CPSNB including neuropathic symptoms and infection could not be evaluated in our meta-analysis because 4 of 5 RCTs enrolled in this meta-analysis have placed a catheter with saline infusion in the single-injection group to enable blinding of patients. Thus we are unable to present comparative results of major and minor complications between CPSNB and single-injection group but rather an overall descriptive data. Therefore, future studies can be designed to place an emphasis in determining the incidence difference of complications between CPSNB and single-injection to provide a more comprehensive result.

## Conclusion

This meta-analysis revealed that continuous popliteal sciatic nerve block can lead to better pain relief for patients who had undergone foot and ankle surgery. Both methods appear to be safe as none of the patients experienced neuropathic symptoms or infection. Further studies with larger sample size are needed to compare the risk of major complications between the two methods.

## Supplementary information

**Additional file 1: Figure S1.** Trial sequential analysis for the effect of continuous popliteal sciatic nerve block (CPSNB) on VAS score at 24 h as compared with the single-injection group. The lower half of the graph below the 0 axis represents the area of VAS score at 24 h in SPSNB, and the upper half represents the VAS score at 24 h in CPSNB. Solid lines (Brown) at + 1.96 and − 1.96 on Y axis represent the conventional model boundaries for TSA with an α of 5%. The required information size (IS) for the conventional boundary model is 50 (shown on X-axis). The red lines represent the α-spending boundary (upper O’Brien-Fleming with α of 5% and β of 20%). The area to the left of the wedge formed by the red lines of α-spending is the area of futility. The area to the right of the wedge is the area of equivalence. The RIS for α-spending boundary model is 66 (shown on vertical line intersecting X-axis in red) Cumulative Z scores fall within the superior range for both conventional boundary and the α-spending boundary of the O’Brien-Fleming model.

**Additional file 2: Figure S2.** Trial sequential analysis for the effect of continuous popliteal sciatic nerve block (CPSNB) on VAS score at 48 h as compared with the single-injection group. The lower half of the graph below the 0 axis represents the area of VAS score at 48 h in SPSNB, and the upper half represents the VAS score at 48 h in CPSNB. Solid lines (Brown) at + 1.96 and − 1.96 on Y axis represent the conventional model boundaries for TSA with an α of 5%. The required information size (IS) for the conventional boundary model is 50 (shown on X-axis). The red lines represent the α-spending boundary (upper O’Brien-Fleming with α of 5% and β of 20%). The area to the left of the wedge formed by the red lines of α-spending is the area of futility. The area to the right of the wedge is the area of equivalence. The RIS for α-spending boundary model is 76 (shown on vertical line intersecting X-axis in red) Cumulative Z scores fall within the superior range for both conventional boundary and the α-spending boundary of the O’Brien-Fleming model.

**Additional file 3: Figure S3.** Trial sequential analysis for the effect of continuous popliteal sciatic nerve block (CPSNB) on VAS score at 72 h as compared with the single-injection group. The lower half of the graph below the 0 axis represents the area of VAS score at 72 h in SPSNB, and the upper half represents the VAS score at 72 h in CPSNB. Solid lines (Brown) at + 1.96 and − 1.96 on Y axis represent the conventional model boundaries for TSA with an α of 5%. The required information size (IS) for the conventional boundary model is 208 (shown on X-axis). The red lines represent the α-spending boundary (upper O’Brien-Fleming with α of 5% and β of 20%). The area to the left of the wedge formed by the red lines of α-spending is the area of futility. The area to the right of the wedge is the area of equivalence. The RIS for α-spending boundary model is 241 (shown on vertical line intersecting X-axis in red). Cumulative Z score didn’t reach the boundary of RIS which implicated inconclusive result.

**Additional file 4: Figure S4.** Trial sequential analysis for the effect of continuous popliteal sciatic nerve block (CPSNB) on oral analgesics consumption at 72 h as compared with the single-injection group. The lower half of the graph below the 0 axis represents the area of oral analgesics consumption at 72 h in SPSNB, and the upper half represents the oral analgesics consumption at 72 h in CPSNB. Solid lines (Brown) at + 1.96 and − 1.96 on Y axis represent the conventional model boundaries for TSA with an α of 5%. The required information size (IS) for the conventional boundary model is 158 (shown on X-axis). The red lines represent the α-spending boundary (upper O’Brien-Fleming with α of 5% and β of 20%). The area to the left of the wedge formed by the red lines of α-spending is the area of futility. The area to the right of the wedge is the area of equivalence. The RIS for α-spending boundary model is 649 (shown on vertical line intersecting X-axis in red). Cumulative Z score didn’t reach the boundary of RIS which implicated inconclusive result.

**Additional file 5: Figure S5.** Trial sequential analysis for the effect of postoperative admission as compared with the single-injection group. The lower half of the graph below the 0 axis represents the area of oral analgesics consumption at 72 h in SPSNB, and the upper half represents postoperative admission in CPSNB. Solid lines (Brown) at + 1.96 and − 1.96 on Y axis represent the conventional model boundaries for TSA with an α of 5%. The required information size (IS) for the conventional boundary model is 208 (shown on X-axis). The red lines represent the α-spending boundary (upper O’Brien-Fleming with α of 5% and β of 20%). The area to the left of the wedge formed by the red lines of α-spending is the area of futility. The area to the right of the wedge is the area of equivalence. The RIS for α-spending boundary model is 980 (shown on vertical line intersecting X-axis in red). Cumulative Z score didn’t reach the boundary of RIS which implicated inconclusive result.

**Additional file 6: Figure S6.** Funnel plot of VAS score at 24 h.

**Additional file 7: Figure S7.** Funnel plot of VAS score at 48 h.

**Additional file 8: Figure S8.** Funnel plot of VAS score at 72 h.

**Additional file 9: Figure S9.** Funnel plot of total amount of oral analgesics at 72 h.

**Additional file 10: Figure S10.** Funnel plot of patient postoperative admission.

## Data Availability

The information to access the data used in the study, is included within this article.

## Abbreviations

CPSNB: Continuous popliteal sciatic nerve block
OR: Odds ratio
PACU: Post-anesthesia care unit
PRISMA: Preferred reporting items for systematic reviews and meta-analysis
RCT: Randomized controlled trial
SMD: Standardized mean difference
VAS: Visual analog scale
