# Supplementary figures and images for: The efficacy and safety of continuous versus single-injection popliteal sciatic nerve block in outpatient foot and ankle surgery: a systematic review and meta-analysis
Source: BMC Musculoskelet Disord. 2019 Oct 10;20:441. doi: 10.1186/s12891-019-2822-7 (PMC6788034; doi:10.1186/s12891-019-2822-7)

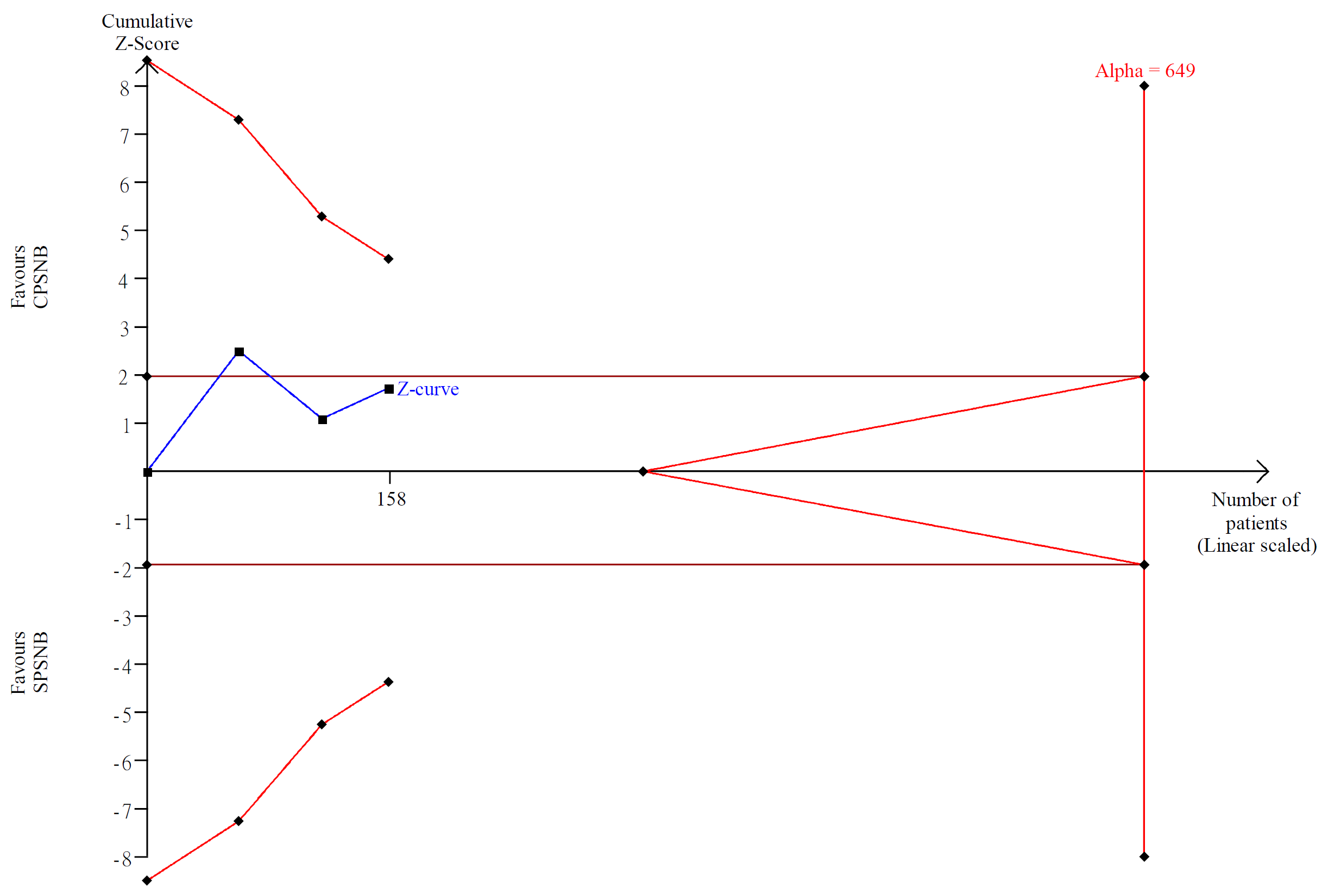

Supplement: Supplementary file 4 — Additional file 4: Figure S4. Trial sequential analysis for the effect of continuous popliteal sciatic nerve block (CPSNB) on oral analgesics consumption at 72 h as compared with the single-injection group. The lower half of the graph below the 0 axis represents the area of oral analgesics consumption at 72 h in SPSNB, and the upper half represents the oral analgesics consumption at 72 h in CPSNB. Solid lines (Brown) at + 1.96 and − 1.96 on Y axis represent the conventional model boundaries for TSA with an α of 5%. The required information size (IS) for the conventional boundary model is 158 (shown on X-axis). The red lines represent the α-spending boundary (upper O’Brien-Fleming with α of 5% and β of 20%). The area to the left of the wedge formed by the red lines of α-spending is the area of futility. The area to the right of the wedge is the area of equivalence. The RIS for α-spending boundary model is 649 (shown on vertical line intersecting X-axis in red). Cumulative Z score didn’t reach the boundary of RIS which implicated inconclusive result. [file 12891_2019_2822_MOESM4_ESM.tif]

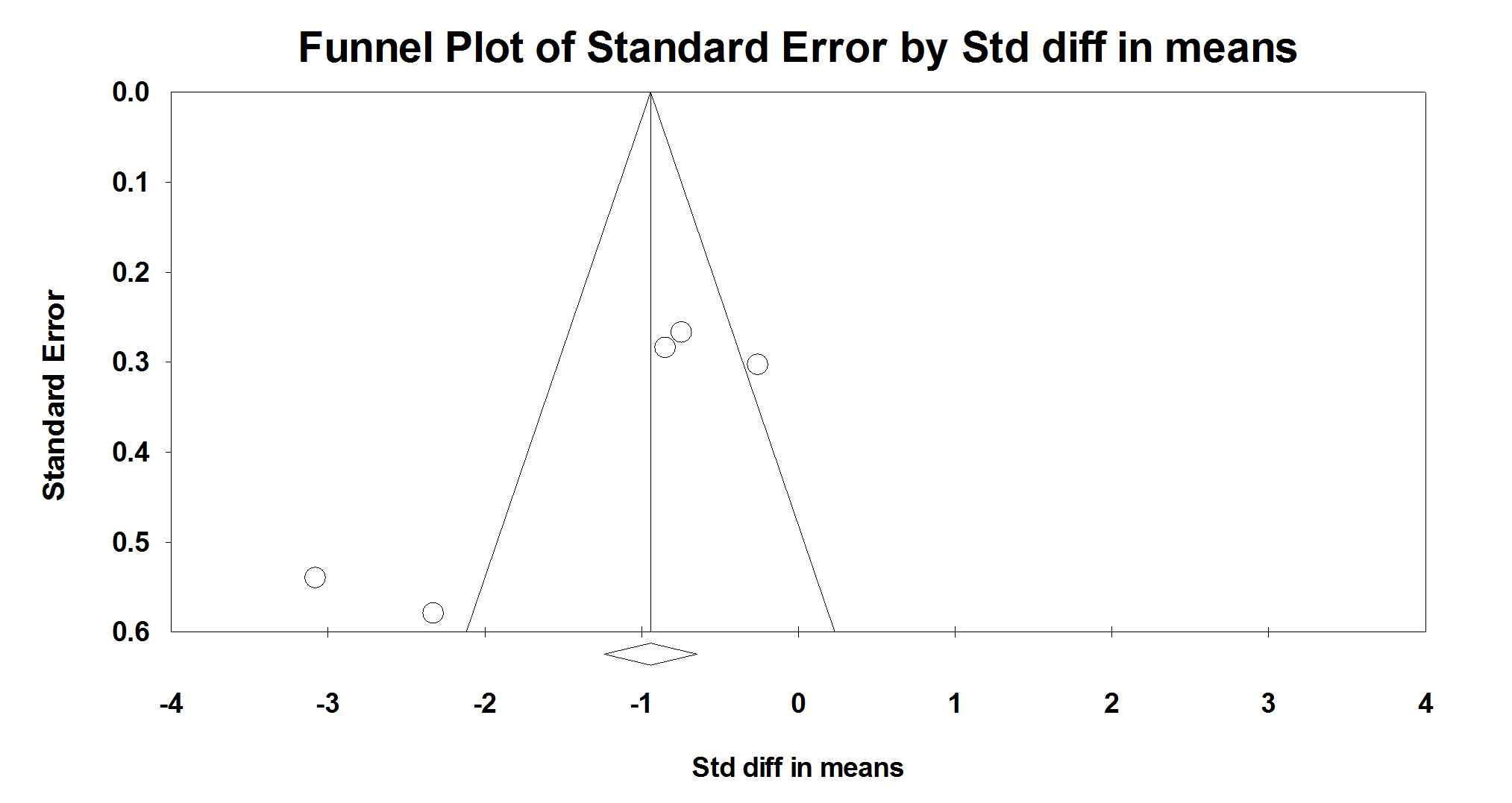

Supplement: Supplementary file 6 — Additional file 6: Figure S6. Funnel plot of VAS score at 24 h. [file 12891_2019_2822_MOESM6_ESM.tif]

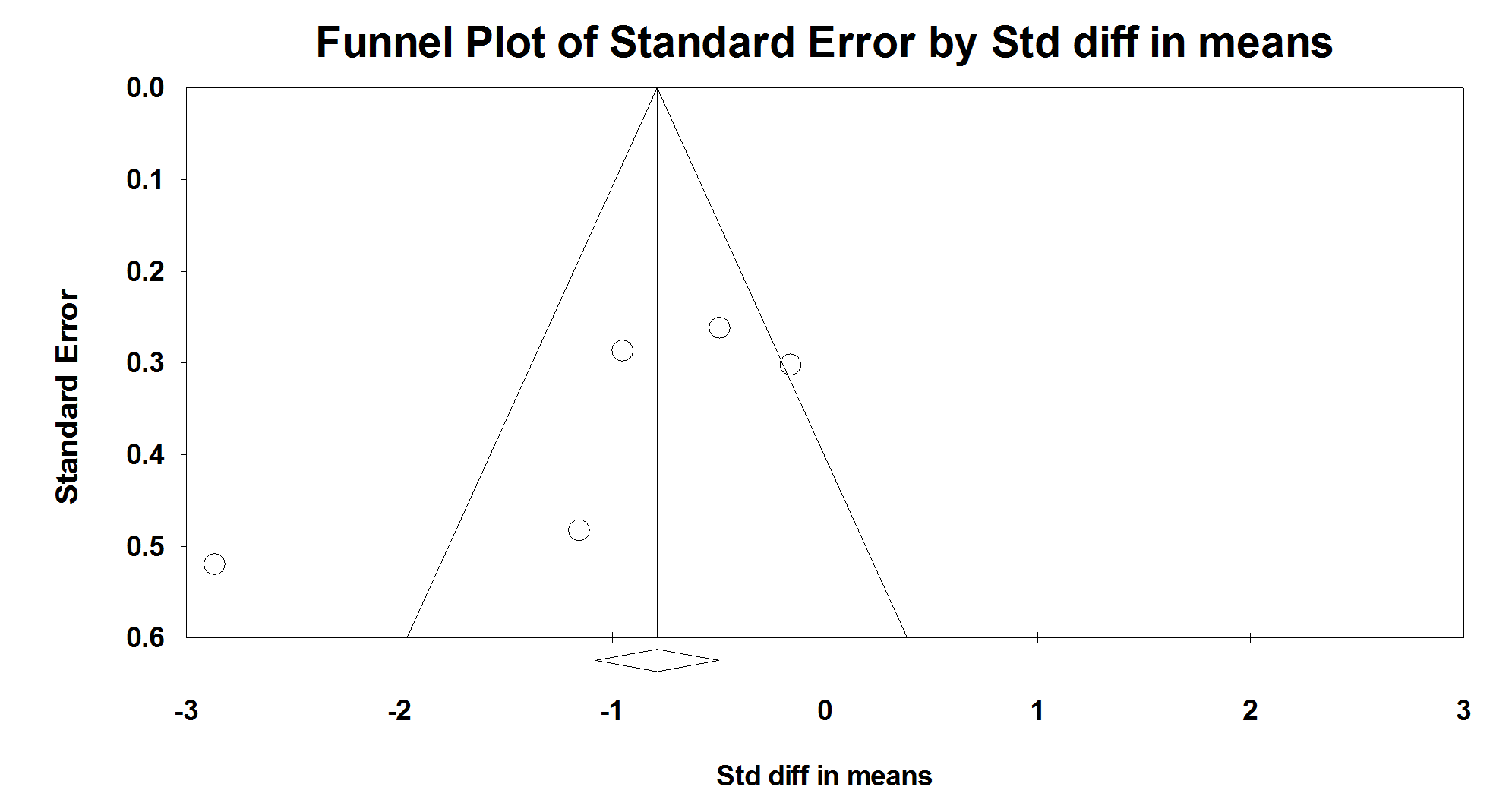

Supplement: Supplementary file 7 — Additional file 7: Figure S7. Funnel plot of VAS score at 48 h. [file 12891_2019_2822_MOESM7_ESM.tif]

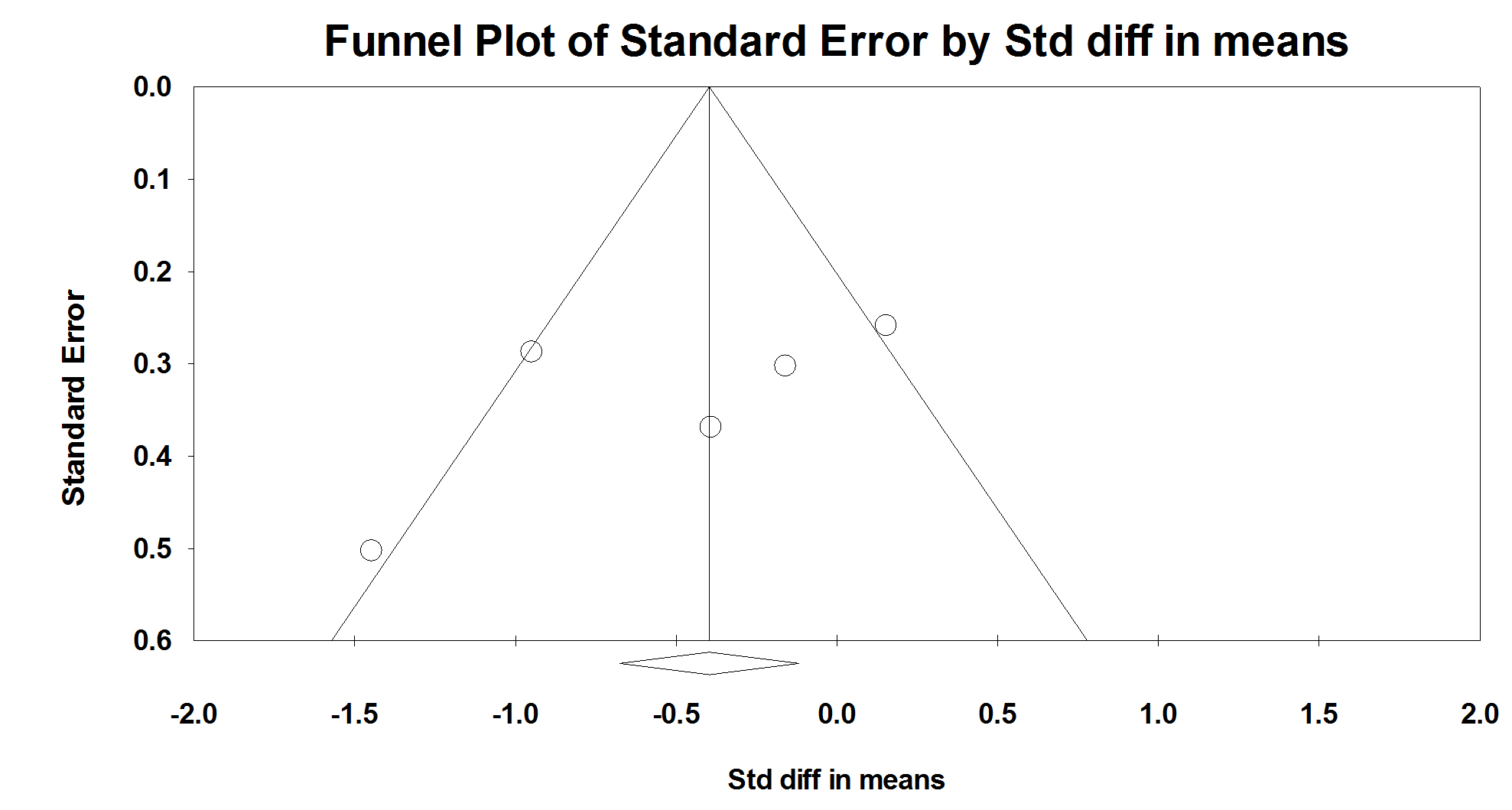

Supplement: Supplementary file 8 — Additional file 8: Figure S8. Funnel plot of VAS score at 72 h. [file 12891_2019_2822_MOESM8_ESM.tif]

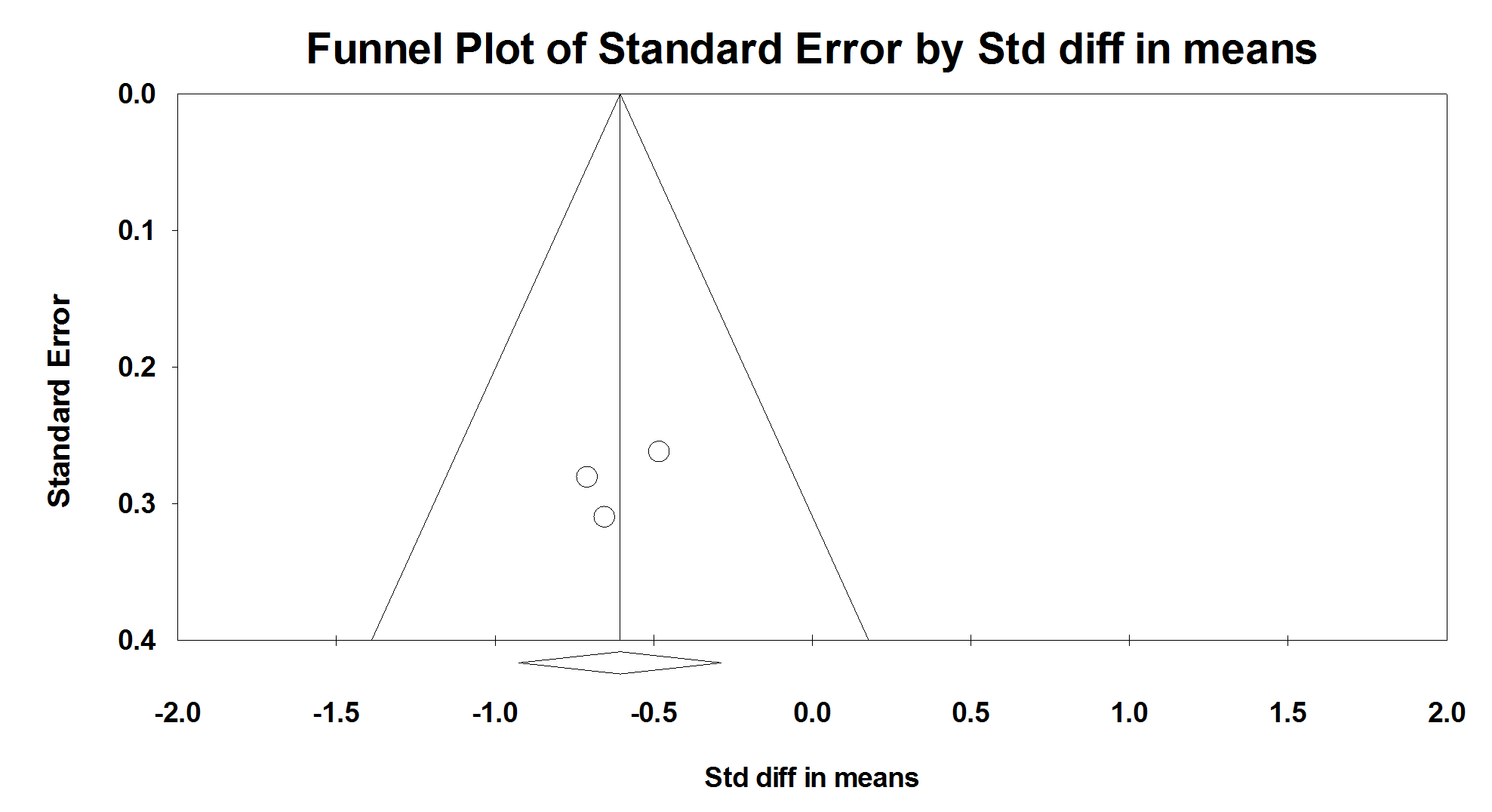

Supplement: Supplementary file 9 — Additional file 9: Figure S9. Funnel plot of total amount of oral analgesics at 72 h. [file 12891_2019_2822_MOESM9_ESM.tif]

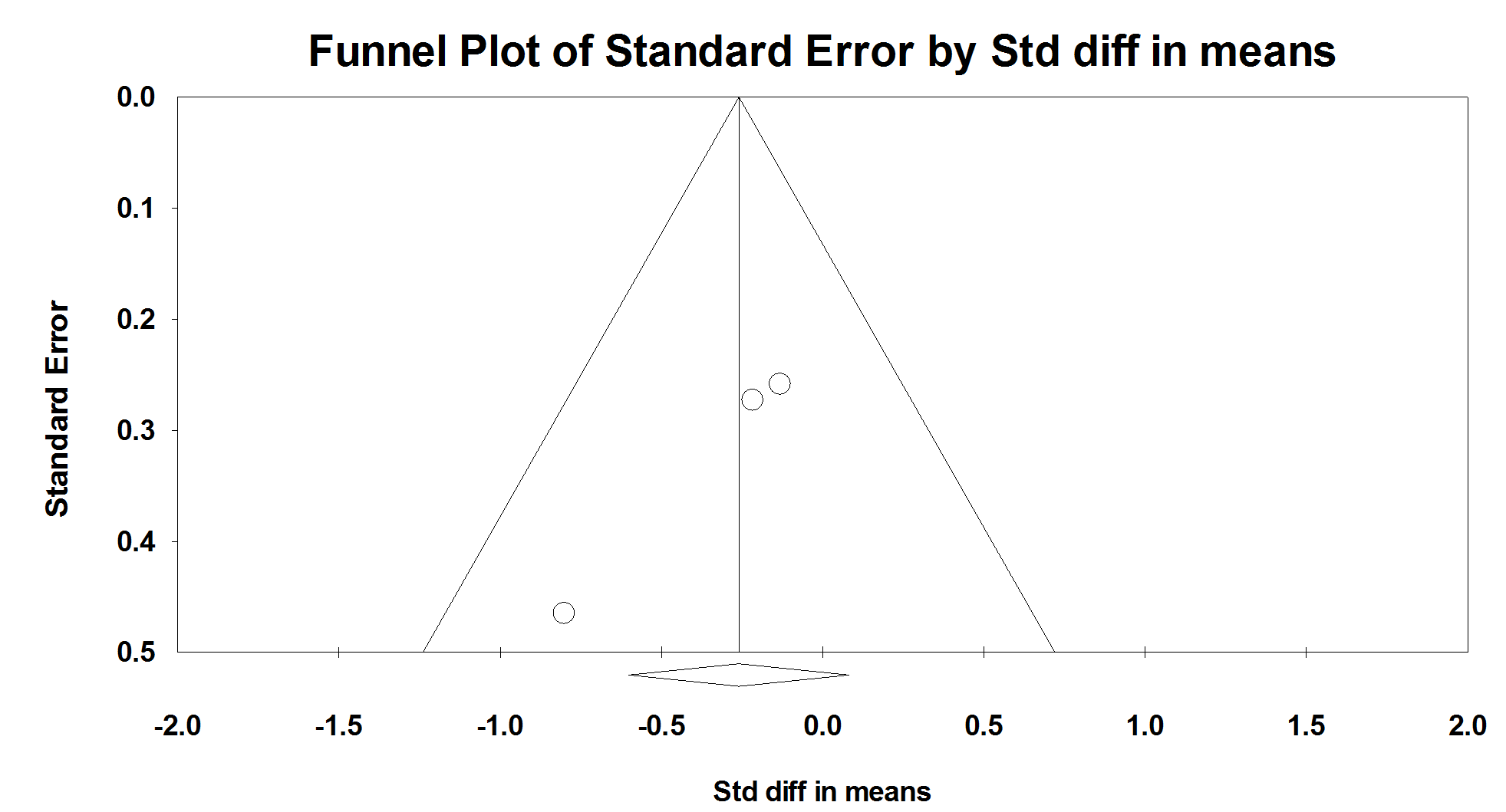

Supplement: Supplementary file 10 — Additional file 10: Figure S10. Funnel plot of patient postoperative admission. [file 12891_2019_2822_MOESM10_ESM.tif]
